# Olive oil in the prevention and management of type 2 diabetes mellitus: a systematic review and meta-analysis of cohort studies and intervention trials

**DOI:** 10.1038/nutd.2017.12

**Published:** 2017-04-10

**Authors:** L Schwingshackl, A-M Lampousi, M P Portillo, D Romaguera, G Hoffmann, H Boeing

**Affiliations:** 1 Department of Epidemiology, German Institute of Human Nutrition, Nuthetal, Germany; 2 Nutrition and Obesity Group, Department of Nutrition and Food Sciences, University of Basque Country (UPV/EHU) and Lucio Lascaray Research Center, Vitoria-Gasteiz, Spain; 3 CIBER Physiopathology of Obesity and Nutrition (CIBERobn), Institute of Health Carlos III, Madrid, Spain; 4 Health Research Institute of Palma (IdISPa), University Hospital Son Espases, Palma de Mallorca, Spain; 5 Department of Nutritional Sciences, Faculty of Life Sciences, University of Vienna, Vienna, Austria

## Abstract

**Background/Objectives:**

Olive oil (OO) as food is composed mainly of fatty acids and bioactive compounds depending from the extraction method. Both had been discussed as health promoting with still open questions. Thus, we conducted a meta-analysis to illustrate the impact of this food on type 2 diabetes (T2D) by investigating the association between OO intake and risk of T2D, and the effect of OO intake in the management of T2D.

**Subjects/Methods:**

Searches were performed in PubMed, Cochrane Library and google scholar. First, we conducted a random effect meta-analysis of prospective cohort studies and trials investigating the association between OO and risk of T2D. Second, a meta-analysis was performed to detect the effects of olive oil on glycemic control in patients with T2D.

**Results:**

Four cohort studies including 15 784 T2D cases and 29 trials were included in the meta-analysis. The highest OO intake category showed a 16% reduced risk of T2D (RR: 0.84; 95% CI: 0.77, 0.92) compared with the lowest. However, we observed evidence for a nonlinear relationship. In T2D patients OO supplementation resulted in a significantly more pronounced reduction in HbA1c (MD: −0.27% 95% CI: −0.37, −0.17) and fasting plasma glucose (MD: −0.44 mmol l^−1^; 95% CI −0.66, −0.22) as compared with the control groups.

**Conclusions:**

This meta-analysis provides evidence that the intake of OO could be beneficial for the prevention and management of T2D. This conclusion regards OO as food, and might not been valid for single components comprising this food.

## Introduction

According to the most recent data by the International Diabetes Federation and the World Health Organization, diabetes represents one of the most important health problems, causing enormous costs, with an estimated prevalence of 350–400 million cases worldwide.^1, 2^ Comprehensive meta-analyses showed significant inverse associations between high adherence to Mediterranean diet and risk of type 2 diabetes (T2D),^3^ and improvements in glycemic control among T2D patients following a Mediterranean diet compared with a low-fat diet.^4^

Extra virgin olive oil is the main source of dietary fat in the Mediterranean diet.^5, 6^ With its high content in monounsaturated fatty acids (MUFA), tyrosol, secoiridoids and lignans (Supplementary Table S1), consumption of extra virgin olive oil might exert beneficial effects in the prevention, development and progression of T2D compared with refined olive oil.^7^ Recent meta-analyses of randomized controlled trials (RCTs) showed consistently that replacing carbohydrates (~5–10% of total energy intake) in general with MUFA as a specific dietary compound has beneficial effects on metabolic risk factors in T2D patients.^8, 9, 10, 11, 12^ In a meta-analysis of 32 cohort studies, we could show that MUFA of mixed animal and vegetable sources *per se* did not yield any significant effects on all-cause mortality and risk of cardiovascular disease, when the top and bottom thirds of baseline dietary fatty acid intake were compared.^13^ However, providing MUFA via olive oil was associated with reduced risk of all-cause mortality, stroke and cardiovascular events.^13^

The promising data from studies regarding olive oil in view of its favorable composition of bioactive compounds motivated us to synthesize the evidence the potential role of olive oil in the prevention and management of T2D. For this purpose, we synthesized data from prospective cohort studies and RCTs investigating the effects of olive oil (administered in either form: for example, olive oil in pure form or as supplement (capsules)) on risk of T2D and markers of glycemic control in patients with T2D.

## Materials and methods

This systematic review was planned and conducted according to the standards of the Meta-analysis of Observational Studies in Epidemiology,^14^ and according to the PRISMA guidelines regarding for RCTs.^15^ Our protocol has been registered in PROSPERO (crd.york.ac.uk/prospero/index.asp, identifier: CRD42016045693). The design of this meta-analysis consisted of two approaches, a meta-analysis on the association between olive oil in the prevention of T2D and a meta-analysis on the effects of olive oil in the management of T2D.

### Literature search and specific data analyses

A systematic search was performed in PubMed, Cochrane Library and google scholar for prospective cohort studies and RCTs published until August 2016. We searched for articles of original research by using the following search terms: (olive oil) AND (diabetes) AND (longitudinal OR prospective OR cohort OR follow-up OR nested OR randomized controlled trial OR randomized OR clinical trials as topic OR placebo OR randomly OR trial). No restrictions to language were made. We manually examined the reference lists from articles eligible for inclusion. The search was conducted independently by two authors (LS and AML), with disagreements resolved by consensus.

### Eligibility criteria

In the first meta-analysis on intake of olive oil and risk of T2D, studies were included if they met the following criteria: (i) studies with a prospective cohort design (including prospective cohort studies, nested case–control studies, RCTs, case–cohort studies); (ii) information of at least one measurement of olive oil intake; (iii) participants aged 18 or older; (iv) considering T2D as outcome (study population had to be free of T2D at the onset of the study).

In the second meta-analysis on the effects of intake of olive oil on parameters of glycemic control studies were included if they met the following criteria:

(i) RCTs with either parallel or crossover design; (ii) intervention with olive oil in pure form (olive oil must be main added fat in the diet; RCTs based on nuts were excluded) or as supplement (capsules) with no other supplementation in the olive oil group; (iii) participants ⩾18 years of age; (iv) enrollment of subjects with T2D;^16^ (v) assessment of the ‘outcome of interest': HbA1c, fasting plasma glucose.

### Data extraction

After determination of the study selection, two reviewers extracted (LS and AML) the following characteristics: the first author's last name, year of publication, study origin, cohort name, sample size, number of cases (only for cohort studies), age at entry, sex, study length, mean baseline BMI/HbA1c and fasting glucose values, outcome(s), outcome assessment, assessment of diet, results, risk estimate (most adjusted measures (hazard ratios (HR), risk ratios (RR) with their corresponding 95% confidence intervals (CIs)) and adjustment factors using our own checklist. When a study provides several risk estimates, the multivariate adjusted model was used. If only separate risk estimates for male and female participants were available in one study, data will be pooled and treated as one study.

### Risk of bias assessment

The Cochrane Collaboration's tool for assessing risk of bias was used to elucidate the risk of bias of the included studies attaching either low, unclear or high risk of bias to the five domains to each study^17^ (Supplementary Figure S1). To assess the risk of bias of the cohort studies, we assessed ascertainment of exposure, assessment of outcome, adequacy of follow-up depending on the outcome, and adjusted basic model and outcome relevant adjustments, based on our own developed tool.^18^

### Statistical analysis

We performed three types of analysis investigating the association between olive oil and risk of T2D:

High vs low intake meta-analysis: summary risk estimated for high vs low intake of olive oil and risk of T2D by applying random effect models.
Dose–response meta-analyses: we investigated the association between intake of dietary factors as a continuous variable and risk of chronic diseases, by performing a dose–response meta-analysis as described by Orsini *et al.* and Greenland and Longnecker.^19, 20^ This method requires for at least three exposure categories: the quantified exposure value and the RRs with the respective 95% CI, as well as the number of cases and person-years.
To examine possible nonlinear associations, we calculated restricted cubic splines for each study with more than three categories of exposure, using three fixed knots at 10, 50, and 90% through the total distribution of the reported intake, and combined them using multivariate meta-analysis.^21^

Investigating the effects of olive oil in the management of glycemic control was done using a random effects model in which the post-intervention values (if not available, we imputed the changes from baseline values, as recommended by the Cochrane Handbook^22^) and corresponding standard deviations of intervention and control/intervention groups were pooled. Pooled effects of the different interventions were investigated as mean difference (MD). Heterogeneity between trial results was tested with a standard *χ*^2^ test. The *I*^2^ parameter was used to quantify any inconsistency: *I*^2^=((*Q*−df))/*Q* × 100%, where *Q* is the *χ*^2^ statistic and df is its degrees of freedom.^23^ An *I*^2^-value of greater than 50% was considered to represent considerable heterogeneity.^24^ In addition, to identify potential sources of heterogeneity, we stratified the meta-analysis by subgroups: age (⩾60 vs <60 years), study design (crossover vs parallel), study length (⩾6 vs <6 months), administration (pure olive oil vs capsules) and extra virgin olive oil (yes vs not applicable).

Potential small-study effects, such as publication bias, were explored using Egger's test and funnel plots,^25^ if at least 10 studies were available, as recommended by the Cochrane Handbook.^26^

Review Manager 5.3 (Nordic Cochrane Center, Copenhagen) and Stata version 14 software (StataCorp, College Station, TX, USA) were used for the statistical analyses.

### Assessment of quality of meta-evidence

To evaluate the meta-evidence for the association between olive oil and risk of T2D as well as parameters of glycemic control we applied the NutriGrade scoring system.^18^ Based on this scoring system, we recommend four categories to judge the meta-evidence: high, moderate, low and very low taking into account the following cutoff points: ⩾8 points (high meta-evidence); 6 to 7.99 points (moderate meta-evidence); 4 to 5.99 (low meta-evidence); and 0 to 3.99 (very low meta-evidence).

## Results

### Selection of studies

The detailed steps of the meta-analysis article search (Supplementary Figure S2) and selection process are given as a flow diagram. Taken together, four cohort studies and 29 RCTs met the inclusion criteria (Supplementary References 1–34), and were included in the quantitative analysis. Twenty-two studies were performed in Europe, eight studies in North America, two studies in Australia/New Zealand and one study in Asia (Supplementary Table S2 and S3).

The study duration varied between 5.7 and 22 years for cohort studies enrolling 183 370 participants, and between 2 weeks and 4.1 years for RCTs enrolling 3698 participants. The mean age ranged between 33 and 67.2 years.

Owing to the different designs of the RCTs, the RCTs were classified in subgroups according to mode of olive oil intervention and controls as follows:

- Olive oil vs low-fat diet;
- Olive oil vs polyunsaturated fatty acids (PUFA)-rich oils;
- Olive oil vs fish oil.

### Meta-analysis on risk of T2D mellitus

Using random effects meta-analyses, we found that the combined association of the use of olive oil was inversely associated with a lower risk of T2D. When the highest olive oil intake category was compared with the lowest intake category we calculated an RR of 0.84 (95% CI: 0.77–0.92, *P*<0.01; *I*^2^=22%) (Table 1; Supplementary Figure S3). The dose–response meta-analysis revealed that each 10 g daily increase in olive oil was associated with a 9% reduced risk of T2D (RR: 0.91; 95% CI: 0.87–0.95; *P*<0.01; *I*^2^=0%) (Supplementary Figure S4). We observed a nonlinear relationship (*P*<0.01) between olive oil intake and risk of T2D. The risk of T2D decreased by 13% with increasing intake of olive oil up to ~15–20 g day^−1^ (Supplementary Figure S5). No benefit for increasing intake is apparent above this value. A sensitivity analysis excluding the SUN cohort study (since olive oil intake was twice as high compared with the EPIC study and the Harvard cohort studies) showed no evidence of a nonlinear relationship (*P*>0.05).

### Meta-analysis on glycemic control

Olive oil interventions resulted in a significantly more pronounced reduction in HbA1c (MD: −0.27% 95% CI −0.37 to −0.17; *P*<0.01; *I*^2^= 0%) as compared with the respective control groups (Supplementary Figure S6). Subgroup analyses showed only a significant effect comparing olive oil intervention with low-fat diets (MD: −0.35% 95% CI −0.48 to −0.23; *P*<0.01; *I*^2^=0%). No significant differences could be observed comparing olive oil interventions vs fish oil and PUFA-rich oils. Stratified analyses for age, study design, study length, administration of olive oil and type of olive oil confirmed the results of the main analysis. A stronger HbA1c reduction was observed in studies with T2D patients <60 years, and by supplementing EVOO, but these subgroup differences were statistically not significant (Supplementary Table S4).

Fasting plasma glucose values were more decreased in T2D in the olive oil intervention groups compared with controls (MD: −0.44 mmol l^−1^; 95% CI −0.66 to −0.22; *P*<0.01; *I*^2^= 26%). With respect to subgroups, comparing olive with fish oil (MD: −0.29 mmol l^−1^; 95% CI −0.54 to −0.04; *P* =0.02; *I*^2^= 0%) and PUFA-rich oils (MD: −0.85 mmol l^−1^; 95% CI −1.35 to −0.35; *P*<0.01; *I*^2^= 0%), changes in fasting glucose were significantly more pronounced in the olive oil groups when compared with their respective controls as well (Supplementary Figure S7). Stratified analyses for age, study design, study length, administration of olive oil and type of olive oil confirmed the results of the main analysis. A stronger HbA1c reduction was observed in studies with supplying olive in pure form compared with capsules, but these differences were statistically not significant (Supplementary Table S4)

### Sensitivity analyses

In trials with a low risk of bias, olive oil was associated with improvements in HbA1c (MD: −0.28; 95% CI: −0.39, −0.17; *I*^2^=0%) and fasting plasma glucose (MD: −0.34; 95% CI: −0.63, −0.04; *I*^2^= 35%) (Table 1).

### Small-study effect

Overall, only two outcomes (HbA1c and fasting glucose) included sufficient studies for a meta-analysis and also allowed inspection of funnel plots. The funnel plots for HbA1c and fasting plasma glucose indicate both moderate to high symmetry (Supplementary Figures S8 and S9).

### NutriGrade

The NutriGrade meta-evidence score for olive oil intake and risk of T2D was low, and for HbA1c and fasting plasma glucose in T2D patients moderate (Table 1).

## Discussion

In the present systematic review, data of 4 cohort studies and 29 RCTs investigating the effects of olive oil-enriched diets on risk of T2D in healthy individuals and parameters of glycemic control in patients with already established T2D were synthesized. The synthesis revealed olive oil intake as being associated with a decreased risk to develop T2D as well as improved glucose metabolism. The magnitude of effect deserve consideration, since a 0.1% decrease in HbA1c would be estimated to a reduction in cardiovascular disease by approximately 7%.^27^ The intervention studies with olive oil used different control groups (low-fat diet, PUFA-rich oils and fish oil) that slightly differed regarding significance of effects in respect to glycosylated hemoglobin or fasting glucose.

In many studies, olive oil has been suspected to exert beneficial effects on health.^7^ It is an integral part of the Mediterranean diet, providing approximately two-thirds of vegetable fats in this kind of nutrition. However, the Mediterranean diet has many components that have been linked with T2D, and thus it is not surprising that this type of diet has been linked with reduced risk of T2D.^3^ The major question is still not well answered which of the components of the Mediterranean diet is worthwhile to be adopted in countries with other dietary traditions, without having a local substitutional food.

Extra virgin olive oil has some components that are not found in other plant oils. Rapeseed oil has a similar fatty acid composition, and is part of the healthy Nordic diet.^28^ Compared with olive oil, rapeseed oil contains higher levels of alpha-linoleic acid and approximately 1% trans-isomers.^29, 30^ Trans-isomers had been linked with unfavorable effects on blood lipids,^30^ and alpha-linolenic acid is much more reactive for oxidation than oleic acid.^31^ On the other hand, plasma phospholipid alpha-linoleic was inversely associated with T2D in the EPIC-Interact study.^32^ Although PUFA-rich oils such as sunflower oil or corn oil improves glycosylated hemoglobin,^33^ LDL-cholesterol and triacylglycerols,^34^ some evidence indicate a range of changes in lipoprotein particle oxidation which may not lower the risk of cardiovascular disease.^35^ A further beneficial component among others is oleuropein, which is responsible for the high resistance to oxidation of extra virgin olive oil.^36^

When mild production methods are used, the resulting extra virgin olive oil contains high amounts of bioactive compounds such as squalene, carotenoids, triterpenoids, phytosterols, tocopherols and also a wide variety of phenolic compounds including secoiridoids (oleuropein) and their phenolic derivates (tyrosol and hydroxytyrosol), flavonoids, (luteolin) and lignans (Supplementary Table S1).

Phenolic compounds in olive oil were associated with increased levels of HDL-cholesterol and in improvements in endothelial function.^36^ Polyphenols might affect glucose metabolism via an inhibition of carbohydrate digestion and absorption, a reduction of glucose release from the liver or a stimulation of glucose uptake in peripheral tissues.^37^ With their antioxidative properties, they might diminish the production of advanced glycosylated end products such as HbA1c.^38^ Analysis of the results of a subgroup of participants of the PREDIMED trial revealed an inverse association between polyphenol excretion and fasting glucose.^39^ Application of oleuropein and hydroxytyrosol (two phenols abundant in olive leaves) as a supplement resulted in enhanced insulin secretion and sensitivity following oral glucose challenge.^40^ Furthermore, olive leaf extracts prepared as tablets yielded diminished levels of fasting glucose and HbA1c.^41^

Another major component of olive oil is oleic acid, a compound which belongs to the class of monounsaturated fatty acids. In a recent meta-analysis of RCTs performed by Qian *et al.*,^8^ reductions in fasting glucose levels were significantly more pronounced following a high-MUFA diet as compared with a regimen high in carbohydrates as well as high-PUFA diets. In contrast to the findings of our own systematic review, the authors did report only a nonsignificant HbA1c-decreasing effect of MUFA diets.^10^ This could be due to variations in study design and a lower number of trials enrolled in their meta-analysis, but also a lack of a biological role of this class of fatty acids for glucose management. However, improvements in parameters of glycemic control following high-MUFA diets could be confirmed in other studies as well.^9^ As potential mechanism of action, reductions in glycemic load (especially when replacing carbohydrates with MUFA) and the consecutive attenuation in insulin secretion as well as increased insulin sensitivity may explain the beneficial effects of MUFA on glycemic control.^42, 43^ Although there is some evidence of a beneficial effect of plant-based monounsaturated fatty acids, it is still not clear whether these effects are due phenolic compounds of extra virgin olive oil or the fatty acid composition.

Although several studies included in the present meta-analysis supplemented extra virgin olive oil as the main fat source, some studies (especially in the subgroup comparing olive vs fish oil, and in the EPIC study) provided no information regarding the specific type of olive oil. In these studies olive oil was given often as placebo, and there is high probability that refined olive oil was used. Compared with refined olive oil, extra virgin olive oil contains a fourfold (232 vs 62 mg kg^−1^) amount of phenolic compounds.^44^

There is still a lack of sufficient data regarding the intake of other oils than olive oils such as rapeseed oil. In a recent review Hoffman and Gerber^28^ concluded that rapeseed oil cannot be recommended as equivalent in terms of health benefits compared with extra virgin olive oil.

### Strengths and limitations

When dealing with RCTs and cohort studies in the field of nutritional sciences, one has to face a number of limitations due to study designs. For example, RCTs investigating specific dietary compositions often do not compare against placebo, but rather against other compositions or dietary patterns. Other limitations might be high drop-out rates (making it more laborious to evaluate reasons for drop-out) or poor adherence to a dietary regimen. Another common problem is the heterogeneity in trial designs, for example, with respect to trial length, participant characteristics and type of intervention/control. Thus, in the present systematic review, the number of trial participants ranged between 6 and 215, while length of trials varied between 2 weeks and 4.1 years. Given the usually extended time scope, cohort studies are better suited to investigate nutritional effects on incidence of T2D. However, they are not limitation-free (variations in dietary assessment methods making it difficult to compare actual intake of olive oil, recall bias etc.). Moreover, several of the included studies did not specify the type of olive oil used, limiting the interpretation of the present meta-analysis. Additional limitations include the small number of cohort studies included in the high vs low, linear and nonlinear dose–response meta-analysis. The observed nonlinear association between olive oil and risk of T2D should be interpreted with caution, since the SUN cohort reported twice as high olive oil intakes compared with the other cohort studies. Moreover none of the analyses reached a high meta-evidence score, suggesting that further research may provide important evidence on the confidence and may change the effect estimate.

With respect to markers of glycemic control, meta-analyses could only be performed for HbA1c and fasting glucose. HbA1c is regarded to be a useful tool in monitoring the management of glycemic control, although inter-subject variabilities due to the patient's age and their initial HbA1c values should be taken into account.^45^ Moreover, both parameters might not accurately reflect glycemic variability (short-term fluctuations in glycemia within a day or long-term variations within weeks or months). Glycemic variability is supposed to be an independent predictor of diabetic complications.^46^ Data calculated from long-term blood glucose measurements such as standard deviations of blood glucose or the area under the curve for 24 h exposure to glucose are more suitable to assess glycemic variability as compared with HbA1c and fasting glucose.^47^ However, these data were not available for the present systematic review.

The strength of this systematic review is the fact that the available evidence on the effects of olive oil on T2D and glycemic control from both cohort studies and RCTs is synthesized. Moreover, the recently established NutriGrade tool was applied in order to assess the meta-evidence of the respective data in spite of the limitations of prospective studies.^18^

## Conclusion

The present systematic review and meta-analysis provides evidence of favorable effects of olive oil on T2D risk and parameters of glycemic control. In light of other benefits, especially reported for extra virgin olive oil as an integral part of a Mediterranean diet, this vegetable oil represents a suitable component of a balanced diet.

## Figures and Tables

**Table 1:** Pooled estimates of effect sizes (95% confidence intervals) expressed as risk ratio (RR) and mean differences (MD) for the effects of olive oil in the prevention and management of type 2 diabetes mellitus

*Comparison*	*No. of studies*	*No. of cases*	*RR*	*95% CI*	*I*^*2*^*(%) (95% CI)*	*Quality of meta-evidence (NutriGrade)*
*Risk of diabetes*						
High vs low olive oil	5	19 081	0.84	0.77, 0.92	22 (0–67)	Low
Per 10 g daily increase	4	18 900	0.91	0.87, 0.95	0 (0–79)	
*Comparison*	*No of studies*	*Sample size*	*MD*	*95% CI*	I^*2*^*(%) (95% CI)*	
*HbA1c (%)*						
Olive oil vs control	22	1428	−0.27	−0.37, −0.17	0 (0, 45)	Moderate
Low risk of bias trials	16	1186	−0.28	−0.39, −0.17	0	
Olive oil vs LF	8	480	−0.35	−0.48, −0.23	0	
Olive oil vs fish oil	13	908	−0.08	−0.26, 0.10	0	
Olive oil vs PUFA-rich oils	2	40	−0.20	−0.92, 0.51	0	

*Fasting glucose (mmol l*^−1^*)*						
Olive oil vs control	25	1724	−0.44	−0.66, −0.22	26 (0, 54)	Moderate
Low risk of bias trials	17	1444	−0.34	−0.63, −0.05	35	
Olive oil vs LF	8	602	−0.38	−0.84, 0.08	55	
Olive oil vs fish oil	14	1048	−0.29	−0.54, −0.04	0	
Olive oil vs PUFA-rich oils	4	74	−0.85	−1.35, −0.35	0	

Abbreviation: LF, low fat.
