# Supplementary material for: Olive oil in the prevention and management of type 2 diabetes mellitus: a systematic review and meta-analysis of cohort studies and intervention trials
Source: Nutr Diabetes. 2017 Apr 10;7(4):e262–. doi: 10.1038/nutd.2017.12 (PMC5436092; doi:10.1038/nutd.2017.12)
Supplement: Supplementary Material [file nutd201712x1.docx]

Supplementary Tables 1-4

Supplementary Figures 1-9

Supplementary References 1-34

|  | Virgin olive oil | Refined olive oil |
| --- | --- | --- |
| MUFA | 73.7% | |
| SFA | 13.5% | |
| PUFA | 8.4% | |
| Hydroxytyrosol | 14.42±3 | 1.74±0.85 |
| Tyrosol | 27.5±4 | 3±1.3 |
| Total simple phenols | 41.9±6.2 | 4.7±2.2 |
| Total secoiridoids | 27.7±6.8 | 9.3±3.8 |
| Lignans | 41.5±4 | 7.3±2.6 |

Supplementary Table 1: Fatty acid composition of olive oil (%) [1], and concentration of total and individual phenolic compounds in olive oil (mg/kg) [2];

| **Reference** | **year** | **country** | **Cohort name** | **sex** | **Sample size** | **Type 2 diabetes cases** | **dietary assesmment** | **outcome assessment** | **adjustment factors** | **age at entry** | **mean age** | **follow up years** | **Results**  **RR (95% CI)** |
| --- | --- | --- | --- | --- | --- | --- | --- | --- | --- | --- | --- | --- | --- |
| Guasch Ferre [3] | 2015 | USA | NHS | women | 59930 | 5738 | validated FFQ | Self-reported/extra questionnaire/National Diabetes Data group criteria | age, ethnicity, ancestry, smoking status, physical activity, family history of diabetes/hypertension/ hypercholesterolemia, multivitamin use, postmenopausal status, menopausal hormone use, quintiles of the aHEI, total energy intake, BMI | 37-65 | 55 | 22 | Highest vs. lowest category: 0.91 (0.81, 1.01) |
| Guasch Ferre [3] | 2015 | USA | NHS II | women | 85157 | 3914 | validated FFQ | Self-reported/extra questionnaire/National Diabetes Data group criteria | age, ethnicity, ancestry, smoking status, physical activity, family history of diabetes/hypertension/ hypercholesterolemia, multivitamin use, postmenopausal status, menopausal hormone use, quintiles of the aHEI, total energy intake, BMI | 26-45 | 36 | 22 | Highest vs. lowest category: 0.87 (0.73, 1.04) |
| Interact [4] | 2011 | Europe | The Interact | Men | 27792 | 11994 | Country specific validated questionnaires | At least two of the following :self-report, linkage to primary or secondary care registers, drug registers, hospital admissions, and mortality data | BMI educational level, physical activity, smoking status, and total calorie intake | NA | <50 | 3.99 million person-years of follow-up | Highest vs. lowest category:  ♀ 0.80 (0.66, 0.97)  ♂ 0.82 (0.68, 0.99) |
| Mari-Sanchis [5] | 2011 | Spain | SUN | both | 10491 | 42 | SFFQ | diagnosis of diabetes diagnosed by a doctor, patients were asked to confirm diagnosis with additional confirmation questionnaires and their medical records | physical activity, smoking, gestational diabetes, hypercholesterolemia, hypertension, energy intake, family history of diabetes, alcohol intake, trans fat, fruit intake, sugar sweetened beverages, sleep apnea, caffeine intake | 18-75 | 38 | 5.7 | Highest vs. lowest category:  1.11 (0.45, 2.74) |
| Salas Salvado [6] | 2014 | Spain | PREDIMED | both | 2301 | 181 | Intervention | medical records or routine biochemical analysis | age, sex, and body mass index, baseline smoking status, fasting glucose level, prevalence of dyslipidemia and hypertension, energy intake, adherence to MedDiet, physical activity, education level,, and alcohol intake level | 55-80 | 67,2 | 4,1 | Olive oil vs. control:  0.60 (0.43, 0.84) |

Supplementary Table 2: General study characteristics of the included studies investigating the effects of olive oil and risk of type 2 diabetes mellitus

| **Reference** | **Sample size** | **Design** | **Population** | **Mean baseline BMI** | **Mean baseline HbA1c** | **Mean baseline Glucose** | **Mean age** | **Duration** | **Outcome** | **Intervention (Olive Oil)** | **Control** | **Results**  **HbA1c %** ± SD/SE  **Fasting glucose (mmol/l)** ± SD/SE |
| --- | --- | --- | --- | --- | --- | --- | --- | --- | --- | --- | --- | --- |
| Annuzzi 1991 [7] | 8 | Placebo crossover | Type 2 Diabetes | 25.8 | 8.5 | NA | 51 | 2 weeks | Fasting Glucose | olive oil :10g/d | fish oil :10g/d | **Olive oil:** Fasting Glucose: 8.50 ±1.22  **Fish oil** : Fasting Glucose:8.06 ± 1.11 |
| Brynes 2000 [8] | 9 | Crossover | Type 2 Diabetes | 29.8 | MUFA diet: 6.6  PUFA diet: 6.7 | MUFA diet: 8.0  PUFA diet: 7.9 | 56 | 3.5 weeks | HbA1c Fasting Glucose | high-MUFA (olive oil) 20.3 of total energy | high-PUFA(corn oil) 13.4 ± 1.3% of total energy | **MUFA diet**: HbA1c:6.5 ± 0.9 Glucose:8.3 ± 1.8 **PUFA diet**: HbA1c:6.6 ± 0.9 Glucose:8.2 ± 2.1 |
| Connor 1993 [9] | 16 | Placebo crossover | Type 2 Diabetes | 30.6 | 8.8 | 8.9±3.1 | 58.7 | 6 months | HbA1c Fasting Glucose | olive oil :15g/d | fish oil :15g/d | **Olive oil:** HbA1c:9.4± 2. 5 Glucose: 9.55 ± 2.4  **fish oil :** HbA1c:10.1± 3.3 Glucose 9.9 ± 2.9 |
| Esposito 2009 [10] | 215 | Parallel | Type 2 Diabetes | MedDiet: 29.7  low-fat Diet:29.5 | MedDiet: 7.75  low-fat Diet: 7.71 | MedDiet :9.0  low-fat Diet: 8.8 | 52.2 | 48 months | HbA1c  Fasting Glucose | MedDiet (olive oil) 30-50g/d | Low-Fat Diet | **MedDiet**: 48months HbA1c:-0.9 ± 0.6 Glucose: -1.7 ± 1.1 **low fat Diet** : 48months HbA1c:-0.5 ± 0.4 Glucose:-0.8 ± 0.8 |
| Garg 1988 [11] | 10 | Crossover | Type 2 Diabetes | 29 | 11.3 | 7.15 | 56 | 4 weeks | HbA1c  Fasting Glucose | High MUFA: 33% of total energy monosaturated fat (olive oil main fat source) | LF-High CHO 9% of total energy monosaturated fat (corn&palm oil main fat source) | **High MUFA**:  HbA1c :8.1. ±1.58 Glucose:5.6 ± 0.54  **LF-High CHO** : HbA1c:7.8 ±2.21 Glucose:6.49 ± 0.89 |
| Garg 1992 [12] | 8 | Crossover | Type 2 Diabetes | 30 | 8.7 | NA | 63 | 3 weeks | HbA1c Fasting Glucose | Low-CHO-High fat 32% of total energy monosaturated fat  (olive oil main fat source) | High CHO  12% of total energy monosaturated fat  (cornpalm oil main fat source) | **LowCHO-High fat**: HbA1c:7.1 ±1.98 Glucose:7.6 ± 2.26 **HighCHO** : HbA1c:7.8 ± 2.26 Glucose:7.7 ± 2.26 |
| Garg 1994 [13] | 51 | Crossover | Type 2 Diabetes | 28.1 | NA | NA | 58 | 6 weeks | Fasting Glucose | high MUFA (olive oil) 25% of total energy | high CHOolive oil 10% total energy | **High MUFA (last 3 days)**:  Glucose:8.1 ± 1.8  **High-CHO (last 3 days)**:  Glucose:8.3 ± 1.9 |
| Hendra 1990 [14] | 80 | Placebo parallel | Type 2 Diabetes | NA | NA | Olive oil: 10.8 Fish oil: 11.2 | 55.9 | 6 weeks | Fasting Glucose | olive oil :10g/d | fish oil :10g/d | **Olive oil:** Fasting Glucose: 11.3(9.9,12.7) **Fish oil** : Fasting Glucose: 12.5(10.9,14.3) |
| Itsiopoulos 2011 [15] | 27 | Crossover | Type 2 Diabetes | 30.7 | 7.1 | NA | 59 | 12 weeks | HbA1c Fasting Glucose | Cretan Mediterranean 65(55-75)g/d olive oil | Habitual diet 6(2-10)g/d olive oil | **Cretan Mediterranean**: HbA1c: 6.8 (6.3-7.3) Glucose: 8.9 (7.8-10.0) **Habitual diet**: HbA1c: 7.1 (6.5-7.7) Glucose: 9.3 (8.2-10.4) |
| Jensen 1989 [16] | 18 | Crossover | Type 2 Diabetes | NA | 9.5 | 9.9 | 34.5 | 8 weeks | HbA1c Fasting Glucose | Olive oil: 21 ml/d | Cod-liver oil: 21 ml/d | **Olive oil:**  HbA1c: 9.5 ± 0.4  Fasting glucose: 12.6 ± 0.9  **Cod-liver oil:**  HbA1c: 9.6 ± 0.3  Fasting glucose: 11 ± 1.1 |
| Lasa 2014 [17] | 141 | Parallel | Type 2 Diabetes | Mediterranean olive oil : 29.4 LF: 29.8 | NA | Mediterranean olive oil : 147.5  LF: 149.9 | 67.2 | 12 months | Fasting Glucose | Mediterranean (olive oil) n=67 118.2 (45.1) g/d | Mediterranean (nuts) n=74 41.4 (14.8) g olive oil/d,  Low fat n=50  39.9 (15.3) g olive oil/d | **Mediterranean olive oil** : Glucose change:  0.2 ± 2.3  **Low Fat**:  Glucose: -0.15 |
| Lobraico 2015 [18] | 47 | Crossover | Type 2 Diabetes | 30.5 | 7.9 | 144.2 | 64.8 | 4 weeks | HbA1c Fasting Glucose | olive oil (2capsules) | krill oil (2 capsules, 1000mg omega-3) | **Olive oil**: HbA1c:7.7±1.6  Glucose:8.1± 3.18  **Krill oil**: HbA1c:7.6±1.5 Glucose: 7.96 ± 2.72 |
| Madigan 2000 [19] | 11 | Crossover | Type 2 Diabetes | Oleic acid diet: 27.8  Linoleic acid diet: 27.7 | NA | NA | 56 | 2 weeks | Fasting Glucose | olive oil: 30 ml/d | sunflower oil: 30 ml/d | **Olive oil:** Glucose : 7.6 ± 0. 7 **fish oil :** Glucose : 8.5 ± 0.8 |
| Madigan 2005 [20] | 6 | Crossover | Type 2 Diabetes | 28 | 5,2 | NA | 56 | 2 weeks | Fasting Glucose | Oleic acid (olive oil) 30mL/d | Linoleic acid (sunflower) 30mL/d | **Oleic acid (olive oil)** Glucose:7.8±1.0 **Linoleic acid(sunflower)**; Glucose:9.0±0.9 |
| McGrath 1996 [21] | 23 | Placebo crossover | Type 2 Diabetes | 26.6 | 9.6 | 10.2 | 53 | 6 weeks | HbA1c Fasting Glucose | olive oil :10g/d | fish oil :10g/d | **Olive oil:** HbA1c:9.7 (8.5-10.9) Glucose:11 (9.3-12.7) **Fish oil** : HbA1c:9.9 (8.5-11.3) Glucose: 11.4 (9.7- 13.3) |
| Mc Manus 1996 [22] | 11 | Placebo crossover | Type 2 Diabetes | 28 | 5.8 | 8.0 | 61.8 | 12 weeks | HbA1c Fasting Glucose | olive oil: 35mg 18:1 FA*Kg-1 *day-1 | fish oil : 35mg 20:5 and 22:6 FA*Kg-1 *day-1 (=2.8 ± 0.2 g n-3 FA/day) linseed oil : 35mg 18:3 FA*Kg-1 *day-1 | **Olive oil:** HbA1c: 6.1 ± 1.32 Glucose: 7.6 ± 1.99 **Fish oil** : HbA1c: 6.5 ± 1.99 Glucose: 8.2 ± 2.98 **Linseed oil:** HbA1c:6.6 ± 1.99 Glucose: 7.9 ± 2.65 |
| Parillo 1993 [23] | 10 | Crossover | Type 2 Diabetes | 26.7 | NA | 6.6 | 52.7 | 2 weeks | Fasting Glucose | high-MUFA-low CHO (olive oil) 29% MUFA of total energy 75g olive oil/d | low-MUFA-high CHO  13% MUFA of total energy  20 g olive oil/d | **high-MUFA-low CHO (olive oil):** Glucose: 6.38 ± 1.97 **low-MUFA-high CHO** : Glucose: 6.74 ± 2.28 |
| Patti 1999 [24] | 16 | Placebo controlled Parallel | Type 2 Diabetes | NA | Olive oil:6.9 Fish oil:7.3 | Olive oil: 9.2 Fish oil: 10.2 | 56 | 6 months | HbA1c Fasting Glucose | olive oil capsules: 3g/d (first 2 months) 2g/d last 4 months | fish oil | **Olive oil:** HbA1c: 7.7 ±0.5 Glucose: 10.3 ± 1 **Fish oil** : HbA1c: 8.3±0.5 Glucose: 10.9.±0.5 |
| Rodriguez Villar 2000 [25] | 12 | Crossover | Type 2 Diabetes | 27.9 | 6.4 | 176 | NA | 6 weeks | HbA1c Fasting glucose | high MUFA (olive oil) 55.6 g/d olive oil | high CHO 16.3 g/d olive oil | **High MUFA**: HbA1c:6.7 ± 1.3 Glucose: 9.82 ± 3.2  **HighCHO** : HbA1c:6.5 ± 1.0 Glucose: 9.1 ± 3.2 |
| Rodriguez Villar 2004 [26] | 22 | Crossover | Type 2 Diabetes | 28.3 | 6.5 | 9.0 | 61 | 6 weeks | HbA1c Fasting Glucose | high MUFA (olive oil) 24,9% of total energy | high CHO olive oil 13,6% total energy | **High MUFA**: HbA1c:6.6 ± 0.9 Glucose:9.3 ± 3.2 **HighCHO** : HbA1c:6.5 ± 0.8 Glucose:8.3 ± 2.4 |
| Rossing 1995 [27] | 19 | Placebo parallel | Type 2 Diabetes | Olive oil: 23.2  Fish oil: 24.5 | Olive oil: 9.2  Fish oil: 8.8 | NA | 33 | 12 months | HbA1c | olive oil: 21 ml/d | Cod-liver oil: 21 ml/d | **Olive oil:** HbA1c: 9.5± 0. 2 **fish oil :** HbA1c: 8.8 ± 0.4 |
| Sirtori 1997 [28] | 203 | Placebo controlled | Type 2 Diabetes | NA | Olive oil:7.14 EPA+DHA:7.25 | Olive oil: 8.14 Fish oil: 8.26 | 58.2 | 6 months | HbA1c Fasting Glucose | olive oil : 3 capsules/d (first 2 months) 2 capsules/d (last 4 months) | 1530 mg (EPA) and 1050mg (DHA) /day (first 2 months) 1020 mg EPA and 700 mg DHA/ day (last 4 months) | **Olive oil:** HbA1c:6.89 ±1.42 Glucose:7.93 ±2.05 **EPA+DHA** : HbA1c:7.05 ±1.64 Glucose:8.16±2.05 |
| Stirban 2010 [29] | 34 | Placebo crossover | Type 2 Diabetes | 31.2 | 7.12± 1.11 | 8.3 | 56.8 | 6 weeks | HbA1c Fasting Glucose | olive oil :2g/d | fish oil (EPA/DHA) :2g/d | **Olive oil:** HbA1c:7.1 ± 1.1 Glucose: 8.05 ± 1.9  **EPA+DHA** : HbA1c:7.1 ± 1.1 Glucose 8.0 ± 1.6 |
| Vessby 1990 [30] | 14 | Placebo crossover | Type 2 Diabetes | NA | 8.1± NA | 8.1± NA | 30-72 | 8 weeks | HbA1c Fasting Glucose | olive oil: 10g/d | fish oil (EPA): 10g/d | **Olive oil:**  -0.15 ± NA  -0.51± NA  **Fish oil:**  0.3 ± NA  0.62± NA |
| Westerveld 1993 [31] | 24 | Placebo parallel | Type 2 Diabetes | olive oil: 28.6  EPA 1800mg : 23.5  EPA 900mg : 26.8 | olive oil: 9.2  EPA 1800mg : 8.6  EPA 900mg : 7.6 | NA | 56.5 | 8 weeks | HbA1c | olive oil :1656 mg/d | EPA : 1800mg/d EPA: 900 mg/d | **Olive oil:** HbA1c:9.3 ± 3 **EPA 1800 mg** : HbA1c:7.9 ±2.1 **EPA 900 mg :** HbA1c:8.1 ± 2.8 |
| Wong 2010 [32] | 97 | Placebo parallel | Type 2 Diabetes | Olive oil: 26.4  Fish oil:25.2 | Olive oil: 7.5 Fish oil: 7.9 | Olive oil: 7.2  Fish oil: 8.1 | 60.1 | 12 weeks | Fasting Glucose change | olive oil :4g/d | fish oil :4g/d | **Olive oil:** Fasting Glucose:-0.30 ± 1.13 **Fish oil** : Fasting Glucose: -0.17 ± 1.69 |
| Woodman 2002 [33] | 59 | Placebo controlled Parallel | Type 2 Diabetes | EPA : 27.9  DHA: 30.6  olive oil: 29.9 | EPA : 7.14 ± DHA: 7.48  Combined (EPA+DHA): 7.31 olive oil: 7.14 | EPA : 7.46  DHA: 8.25  Combined (EPA+DHA): 7.87  olive oil: 7.96 | 61,2 | 6 weeks | HbA1c Fasting Glucose | olive oil : 1 capsule/d (4g) | EPA: 1 capsule/d (4g) DHA: 1 capsule/d (4g) | **Olive oil:** HbA1c:7.04 ± 0.6 Glucose:7.55 ± 1.36 **EPA:** HbA1c:7.21 ± 1.07 Glucose:8.49 ± 2.19 **DHA:** HbA1c:7.33 ± 0.85 Glucose:8.80 ± 1.23 **Combined (EPA+DHA)** HbA1c:7.27 ± 0.95 Glucose:8.65± 1.74 |
| Zeman 2006 [34] | 24 | Crossover | Type 2 Diabetes | NA | 7.75± | 9.4 | NA | 12 weeks | HbA1c,  Fasting glucose | olive oil: 3.6 g/d | PUFA n-3: 3.6 g/d | **Olive oil:** HbA1c: 9.7 ± 0.8  Glucose : 7.6 ± 0.8  **PUFA n-3:** HbA1c: 7.53 ± 1.69  Glucose : 9.9 ± 0. 7 |

Supplementary Table 3: General study characteristics of the included studies investigating the effects of olive oil on glycemic control in patients with type 2 diabetes mellitus

| **Comparison** | **No of studies** | **Sample size** | **MD** | **95% CI** | **I^2^ (%)** | **Test for subgroup difference** |
| --- | --- | --- | --- | --- | --- | --- |
| **HbA1c (%)** | | | | | | |
| **Age** | | | | | | |
| < 60 years | 14 | 1084 | -0.31 | [-0.42, -0.20] | 0 | 0.13 |
| ≥ 60 years | 7 | 296 | -0.11 | [-0.34, 0.11] | 0 |  |
| **Study design** | | | | | | |
| Parallel | 7 | 772 | -0.21 | [-0.45, 0.03] | 41 | 0.45 |
| Cross-over | 15 | 656 | -0.09 | [-0.29, 0.11] | 0 |  |
| **Study length** | | | | | | |
| < 6 months | 16 | 699 | -0.10 | [-0.29, 0.08] | 0 | 0.40 |
| ≥ 6 months | 6 | 729 | -0.24 | [-0.49, 0.02] | 37 |  |
| **Administration of Olive oil** | | | | | | |
| Pure olive oil | 10 | 540 | -0.19 | [-0.43, 0.05] | 27 | 0.68 |
| Supplements (capsules) | 12 | 888 | -0.13 | [-0.31, 0.06] | 0 |  |
| **Extra virgin olive oil** | | | | | | |
| Yes | 5 | 360 | -0.26 | [-0.49, -0.02] | 21 | 0.36 |
| Not applicable | 17 | 1068 | -0.12 | [-0.29, 0.04] | 0 |  |
|  | | | | | | |
| **Fasting glucose (mmol/l)** | | | | | | |
| **Age** | | | | | | |
| < 60 years | 16 | 1097 | -0.57 | [-0.84, -0.30] | 26 | 0.15 |
| ≥ 60 years | 8 | 603 | -0.26 | [-0.57, 0.04] | 0 |  |
| **Study design** | | | | | | |
| Parallel | 7 | 1014 | -0.52 | [-0.89, -0.15] | 60 | 0.63 |
| Cross-over | 18 | 710 | -0.41 | [-0.68, -0.14] | 0 |  |
| **Study length** | | | | | | |
| < 6 months | 20 | 906 | -0.47 | [-0.70, -0.24] | 0 | 0.82 |
| ≥ 6 months | 5 | 818 | -0.40 | [-0.92, 0.12] | 70 |  |
| **Administration of Olive oil** | | | | | | |
| Pure olive oil | 11 | 549 | -0.65 | [-0.97, -0.33] | 26 | 0.05 |
| Supplements (capsules) | 14 | 1175 | -0.25 | [-0.49, -0.02] | 0 |  |
| **Extra virgin olive oil** | | | | | | |
| Yes | 5 | 478 | -0.15 | [-0.93, 0.64] | 71 | 0.48 |
| No | 20 | 1246 | -0.44 | [-0.64, -0.23] | 0 |  |

Supplementary Table 4: Pooled estimates of effect sizes (95% confidence intervals) expressed as mean differences (MD) for the effects of olive oil in the management of type 2 diabetes mellitus, stratified by age, study design, study length, administration of olive oil, and extra virgin olive oil

Supplementary Figure 1: Risk of bias across the included intervention trials


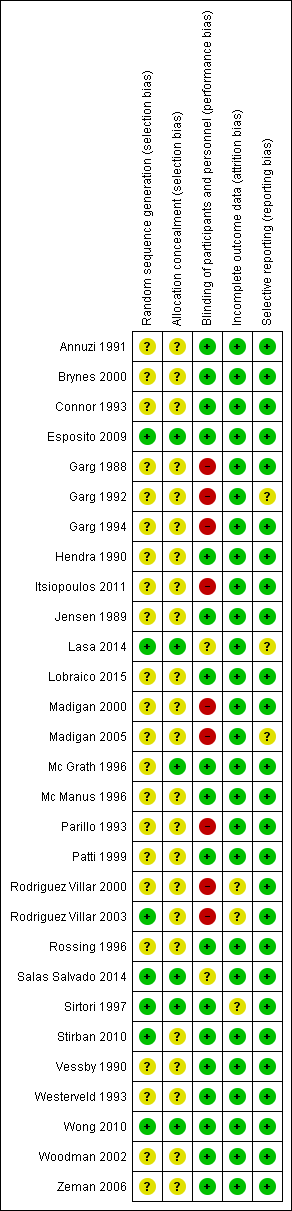


Records identified through database searching: (until September 14^th^, 2016)

PubMed (n=145)

Central (n=88)

Additional records identified through other sources (handpicking) (n=53)

Records screened after excluding duplicates (n=213)

Records excluded after title/abstract screening: not about survival or recurrence, not cohort studies, exposure not relevant

(n= 169)

Full-text articles assessed for eligibility

(n=44)

Full-text articles excluded, with reasons (n=11)

- Intervention group: Olive oil and nuts (n=2)
- Intervention group: Olive oil and corn oil (n=1)
- Intervention group: Olive oil and rapeseed oil (n=1)
- Intervention and control group: both supplemented with olive oil (n=1)
- RCTs based on a test meal (n=3)
- Type 1 diabetes mellitus patients (n=2)
- Gestational diabetes mellitus patients (n=1)

Studies included in qualitative and quantitative synthesis

(n=33)

Cohort studies (n=4)

RCTs (n=29)

Supplementary Figure 2: Flow diagram


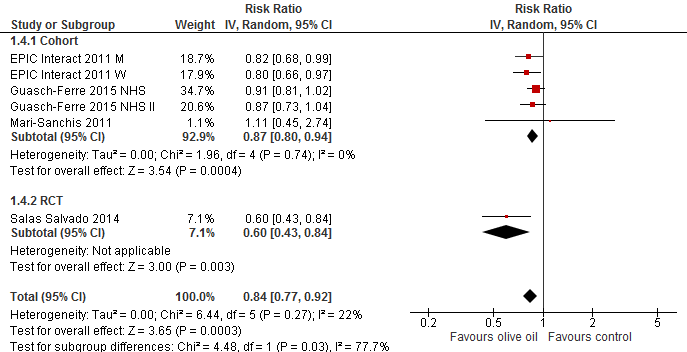


Supplementary Figure 3: Forest plot showing dose-response risk ratio (RR) with 95% CI for the highest vs. lowest olive oil intake category on risk of type 2 diabetes


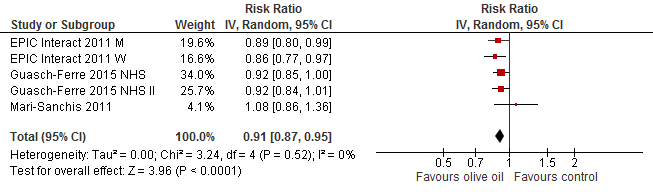
 Supplementary Figure 4: Forest plot showing dose-response risk ratio (RR) with 95% CI for 10g/daily increase in olive oil on risk of type 2 diabetes

p<0.01

Supplementary Figure 5: Non-linear dose-response relationship between olive oil (g/daily) intake and risk of type 2 diabetes


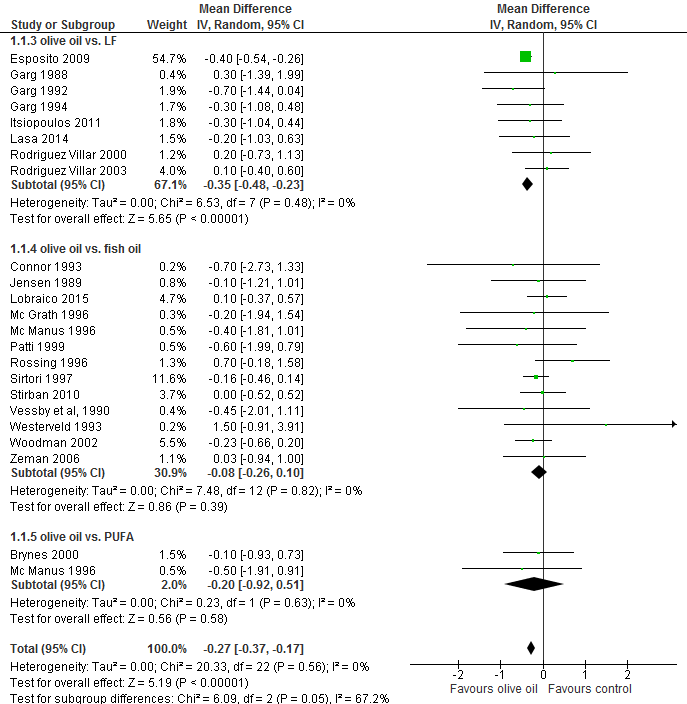


Supplementary Figure 6: Forest plot showing pooled mean difference (MD) with 95% CI for glycosylated hemoglobin (%) comparing olive oil interventions vs control groups.


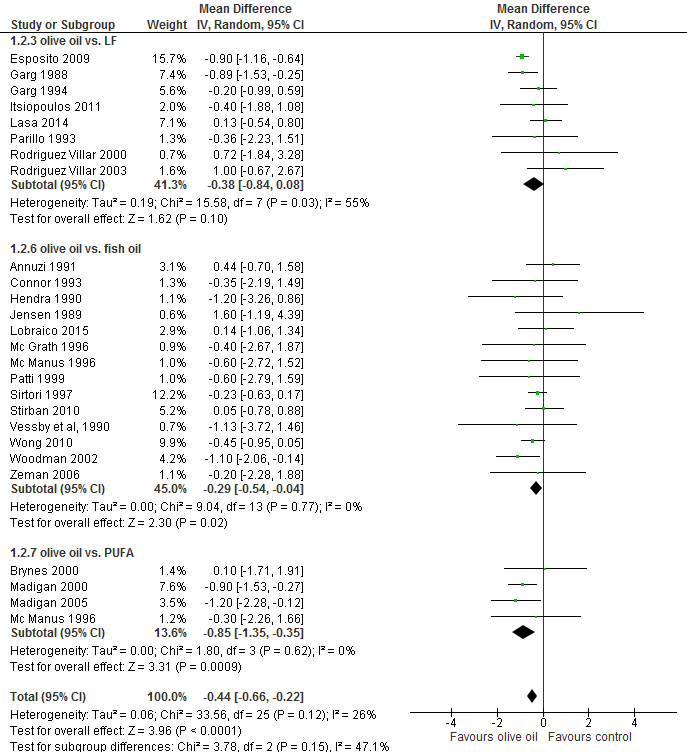


Supplementary Figure 7: Forest plot showing pooled mean difference (MD) with 95% CI for fasting glucose (mmol/l) comparing olive oil interventions vs control groups.


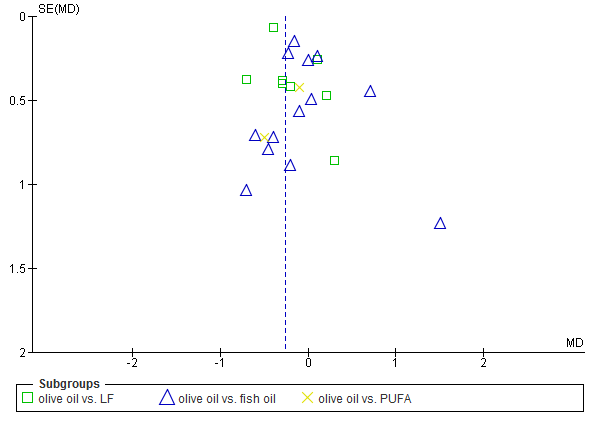


Supplementary Figure 8: Funnel plot showing study precision against the mean difference with SE for olive oil intervention and glycosylated hemoglobin.


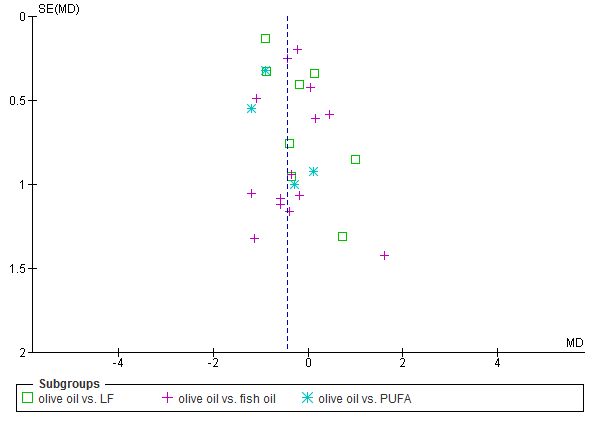


Supplementary Figure 9: Funnel plot showing study precision against the mean difference with SE for olive oil intervention and fasting plasma glucose.

Supplementary References

[1-34]

[1] Schwingshackl L, Hoffmann G (2012) Monounsaturated fatty acids and risk of cardiovascular disease: synopsis of the evidence available from systematic reviews and meta-analyses. Nutrients 4: 1989-2007

[2] Owen RW, Mier W, Giacosa A, Hull WE, Spiegelhalder B, Bartsch H (2000) Phenolic compounds and squalene in olive oils: the concentration and antioxidant potential of total phenols, simple phenols, secoiridoids, lignansand squalene. Food and chemical toxicology : an international journal published for the British Industrial Biological Research Association 38: 647-659

[3] Guasch-Ferre M, Hruby A, Salas-Salvado J, et al. (2015) Olive oil consumption and risk of type 2 diabetes in US women. Am J Clin Nutr 102: 479-486

[4] Romaguera D, Guevara M, Norat T, et al. (2011) Mediterranean diet and type 2 diabetes risk in the European Prospective Investigation into Cancer and Nutrition (EPIC) study: the InterAct project. Diabetes Care 34: 1913-1918

[5] Mari-Sanchis A, Beunza JJ, Bes-Rastrollo M, et al. (2011) [Olive oil consumption and incidence of diabetes mellitus, in the Spanish sun cohort]. Nutr Hosp 26: 137-143

[6] Salas-Salvado J, Bullo M, Estruch R, et al. (2014) Prevention of diabetes with Mediterranean diets: a subgroup analysis of a randomized trial. Ann Intern Med 160: 1-10

[7] Annuzzi G, Rivellese A, Capaldo B, et al. (1991) A controlled study on the effects of n-3 fatty acids on lipid and glucose metabolism in non-insulin-dependent diabetic patients. Atherosclerosis 87: 65-73

[8] Brynes AE, Edwards CM, Jadhav A, Ghatei MA, Bloom SR, Frost GS (2000) Diet-induced change in fatty acid composition of plasma triacylglycerols is not associated with change in glucagon-like peptide 1 or insulin sensitivity in people with type 2 diabetes. Am J Clin Nutr 72: 1111-1118

[9] Connor WE, Prince MJ, Ullmann D, et al. (1993) The hypotriglyceridemic effect of fish oil in adult-onset diabetes without adverse glucose control. Annals of the New York Academy of Sciences 683: 337-340

[10] Esposito K, Maiorino MI, Ciotola M, et al. (2009) Effects of a Mediterranean-style diet on the need for antihyperglycemic drug therapy in patients with newly diagnosed type 2 diabetes: a randomized trial. Ann Intern Med 151: 306-314

[11] Garg A, Bonanome A, Grundy SM, Zhang ZJ, Unger RH (1988) Comparison of a high-carbohydrate diet with a high-monounsaturated-fat diet in patients with non-insulin-dependent diabetes mellitus. N Engl J Med 319: 829-834

[12] Garg A, Grundy SM, Unger RH (1992) Comparison of effects of high and low carbohydrate diets on plasma lipoproteins and insulin sensitivity in patients with mild NIDDM. Diabetes 41: 1278-1285

[13] Garg A, Bantle JP, Henry RR, et al. (1994) Effects of varying carbohydrate content of diet in patients with non-insulin-dependent diabetes mellitus. Jama 271: 1421-1428

[14] Hendra TJ, Britton ME, Roper DR, et al. (1990) Effects of fish oil supplements in NIDDM subjects. Controlled study. Diabetes Care 13: 821-829

[15] Itsiopoulos C, Brazionis L, Kaimakamis M, et al. (2011) Can the Mediterranean diet lower HbA1c in type 2 diabetes? Results from a randomized cross-over study. Nutr Metab Cardiovasc Dis 21: 740-747

[16] Jensen T, Stender S, Goldstein K, Holmer G, Deckert T (1989) Partial normalization by dietary cod-liver oil of increased microvascular albumin leakage in patients with insulin-dependent diabetes and albuminuria. N Engl J Med 321: 1572-1577

[17] Lasa A, Miranda J, Bullo M, et al. (2014) Comparative effect of two Mediterranean diets versus a low-fat diet on glycaemic control in individuals with type 2 diabetes. Eur J Clin Nutr 68: 767-772

[18] Lobraico JM, DiLello LC, Butler AD, Cordisco ME, Petrini JR, Ahmadi R (2015) Effects of krill oil on endothelial function and other cardiovascular risk factors in participants with type 2 diabetes, a randomized controlled trial. BMJ open diabetes research & care 3: e000107

[19] Madigan C, Ryan M, Owens D, Collins P, Tomkin GH (2000) Dietary unsaturated fatty acids in type 2 diabetes: higher levels of postprandial lipoprotein on a linoleic acid-rich sunflower oil diet compared with an oleic acid-rich olive oil diet. Diabetes Care 23: 1472-1477

[20] Madigan C, Ryan M, Owens D, Collins P, Tomkin GH (2005) Comparison of diets high in monounsaturated versus polyunsaturated fatty acid on postprandial lipoproteins in diabetes. Irish journal of medical science 174: 8-20

[21] McGrath LT, Brennan GM, Donnelly JP, Johnston GD, Hayes JR, McVeigh GE (1996) Effect of dietary fish oil supplementation on peroxidation of serum lipids in patients with non-insulin dependent diabetes mellitus. Atherosclerosis 121: 275-283

[22] McManus RM, Jumpson J, Finegood DT, Clandinin MT, Ryan EA (1996) A comparison of the effects of n-3 fatty acids from linseed oil and fish oil in well-controlled type II diabetes. Diabetes Care 19: 463-467

[23] Parillo M, Rivellese AA, Ciardullo AV, et al. (1992) A high-monounsaturated-fat/low-carbohydrate diet improves peripheral insulin sensitivity in non-insulin-dependent diabetic patients. Metabolism 41: 1373-1378

[24] Patti L, Maffettone A, Iovine C, et al. (1999) Long-term effects of fish oil on lipoprotein subfractions and low density lipoprotein size in non-insulin-dependent diabetic patients with hypertriglyceridemia. Atherosclerosis 146: 361-367

[25] Rodriguez-Villar C, Manzanares JM, Casals E, et al. (2000) High-monounsaturated fat, olive oil-rich diet has effects similar to a high-carbohydrate diet on fasting and postprandial state and metabolic profiles of patients with type 2 diabetes. Metabolism 49: 1511-1517

[26] Rodriguez-Villar C, Perez-Heras A, Mercade I, Casals E, Ros E (2004) Comparison of a high-carbohydrate and a high-monounsaturated fat, olive oil-rich diet on the susceptibility of LDL to oxidative modification in subjects with Type 2 diabetes mellitus. Diabetic medicine : a journal of the British Diabetic Association 21: 142-149

[27] Rossing P, Hansen BV, Nielsen FS, Myrup B, Holmer G, Parving HH (1996) Fish oil in diabetic nephropathy. Diabetes Care 19: 1214-1219

[28] Sirtori CR, Paoletti R, Mancini M, et al. (1997) N-3 fatty acids do not lead to an increased diabetic risk in patients with hyperlipidemia and abnormal glucose tolerance. Italian Fish Oil Multicenter Study. Am J Clin Nutr 65: 1874-1881

[29] Stirban A, Nandrean S, Gotting C, et al. (2010) Effects of n-3 fatty acids on macro- and microvascular function in subjects with type 2 diabetes mellitus. Am J Clin Nutr 91: 808-813

[30] Vessby B, Boberg M (1990) Dietary supplementation with n-3 fatty acids may impair glucose homeostasis in patients with non-insulin-dependent diabetes mellitus. Journal of internal medicine 228: 165-171

[31] Westerveld HT, de Graaf JC, van Breugel HH, et al. (1993) Effects of low-dose EPA-E on glycemic control, lipid profile, lipoprotein(a), platelet aggregation, viscosity, and platelet and vessel wall interaction in NIDDM. Diabetes Care 16: 683-688

[32] Wong CY, Yiu KH, Li SW, et al. (2010) Fish-oil supplement has neutral effects on vascular and metabolic function but improves renal function in patients with Type 2 diabetes mellitus. Diabetic medicine : a journal of the British Diabetic Association 27: 54-60

[33] Woodman RJ, Mori TA, Burke V, Puddey IB, Watts GF, Beilin LJ (2002) Effects of purified eicosapentaenoic and docosahexaenoic acids on glycemic control, blood pressure, and serum lipids in type 2 diabetic patients with treated hypertension. Am J Clin Nutr 76: 1007-1015

[34] Zeman M, Zak A, Vecka M, Tvrzicka E, Pisarikova A, Stankova B (2006) N-3 fatty acid supplementation decreases plasma homocysteine in diabetic dyslipidemia treated with statin-fibrate combination. The Journal of nutritional biochemistry 17: 379-384
